# Prevalence of Metabolic Syndrome in Type 2 Diabetes Mellitus Using NCEP-ATPIII, IDF and WHO Definition and Its Agreement in Gwalior Chambal Region of Central India

**DOI:** 10.5539/gjhs.v5n6p142

**Published:** 2013-09-17

**Authors:** Dhananjay Yadav, Sunil Mahajan, Senthil K. Subramanian, Prakash Singh Bisen, Choon Hee Chung, GBKS Prasad

**Affiliations:** 1 School of studies in Biochemistry, Jiwaji University Gwalior (Madhya Pradesh), India; 2 Department of Internal Medicine, Yonsei University Wonju College of Medicine, Wonju, South Korea

**Keywords:** diabetes, metabolic syndrome, prevalence, WHO criteria, NCEP-ATPIII criteria, IDF criteria

## Abstract

The aim of study was to determine the prevalence of metabolic syndrome (MetS) in people with type 2 diabetes mellitus (T2DM). National Cholesterol Education Program (NCEP) ATPIII Criteria, International Diabetes Federation and the World Health Organization (WHO) definitions were used in quantifying the metabolic syndrome and also the concordance between these three criteria’s used for identifying metabolic syndrome.

**Methods:**

This cross-sectional study involved 700 type 2 diabetic subjects from the urban areas of Gwalior Chambal region (Central India). Subjects in the age group of 28-87 yrs were included in the study. Type I diabetics, pregnant ladies and those with chronic viral and bacterial infections and serious metabolic disorders were excluded from the study. Fasting blood glucose, Blood lipids (T-cholesterol, triglyceride, HDL-cholesterol) were assessed and anthropometry blood pressure were measured from all the subjects.

**Results:**

The Prevalence of metabolic syndrome was found to be 45.8%, 57.7% and 28% following NCEP-ATPIII Criteria, IDF and WHO definitions, respectively. Using all the three definitions the prevalence was higher in women in all age groups. ATP III and IDF criteria showed good agreement (κ 0.68) compared to ATP III with WHO (κ 0.54) and IDF with WHO (κ 0.34) criteria. Highest prevalence was observed following IDF definition.

**Conclusions:**

A good agreement was observed between ATPIII and IDF criteria. Maximum prevalence of Metabolic syndrome was recorded when IDF criteria was followed. NCEP-ATPIII criteria for the diagnosis of MetS and this criterion reflected equal importance to the every variable and showed a good agreement between the different criteria used.

## 1. Introduction

The metabolic syndrome is defined as a clustering of key cardiovascular risk factors, namely, abdominal obesity, dyslipidemia, hyperglycemia and hypertension in a single individual (Eckel et al., 2005). Gerald Reaven introduced the concept of the syndrome in 1988. Afterwards this constellation of cardiovascular disease (CVD) risk factors has been given a number of names, such as Syndrome X, dysmetabolic syndrome, insulin resistance syndrome and deadly quartet (Zimmet & Alberti, 2005; Wilson et al., 1998). However, till today its’ observational and epidemiological investigation has long been prevented by the absence of internationally accepted criteria for its diagnosis. To defeat this problem, in 1998, Alberti and Zimmet (1998) proposed for the first time a more unified descriptive “definition” for the diagnosis of metabolic syndrome which they called as World Health Organization (WHO) criteria. Besides, the WHO criterion has not been consistently used because of the requirement to measure serum insulin and urinary microalbumin. This problem is overcome by the Third Report of the National Cholesterol Education Program (NCEP) the Adult Treatment Panel III (ATP III) in 2001. This definition uses only simple clinical measurements of waist circumference (WC), fasting plasma glucose (PG), triglyceride (TG) and high density lipoprotein cholesterol (HDL-C) levels as well as blood pressure (BP) (NCEP, 2001). The ATP III criteria is more practical and found to be a better predictor of coronary heart disease (CHD) risk in the US population (Alexander et al., 2002). Unlike WHO criteria (Alberti et al., 1998) microalbuminurea is not required for ATP III criteria. Recently the ATP III definitions for MetS were renewed in which the new cut-off waist circumference for the Asia and Pacific Region and new cut-off for fasting glucose was introduced (Grundy et al., 2005). Recently, International Diabetes Federation (IDF) in 2005 proposed a new world wide definition of the metabolic syndrome (International Diabetes Federation 2005). The above three definitions are the most popular and commonly used for the diagnosis of Metabolic syndrome (Deepa et al., 2007; Clara Kelliny et al., 2008). The main focus of this definition is central obesity defined by waist circumference and has specific cut-off value for different ethnic populations as a mandatory component in MetS definition. Besides, data on the agreement between the definitions of MetS (WHO, IDF and ATP III) in T2DM population is even more diverse, which make the estimation of MetS difficult to those prognosis the T2DM for risk of cardiovascular disease. Until now, there is no specific criteria for defining MetS in type 2 diabetic population specially for India region, So we have examined MetS prevalence as stated above Gwalior-Chambal’ region of India using all three well known (WHO, IDF and ATP III) definitions and also its validity by concordance between the definitions.

## 2. Methodology

A total of 700 type II diabetic subjects who were willing to take part in the study and who had given informed written consent were randomly selected and recruited from those suffering with type-2 Diabetes Mellitus. Data of 504 males and 196 females, from 28 to 87 years of age, from Gwalior were selected from January 2007 to October 2009 in a cross sectional manner. The diabetic subjects were attending the week end diabetic clinic run in the school of studies in Biochemistry at Jiwaji University Gwalior. Information about subject’s age, sex, monthly income, life style, family history of diabetes and other diseases/disorders were recorded. Height, weight and waist circumferences were measured with the subject barefooted and lightly dressed. The abdominal circumference (waist) was measured at the end of expiration, by wrapping the tape at the level of the umbilicus. Body mass index (BMI) was calculated as weight in kilograms divided by the square of height in meters. Blood pressure was measured with special precaution to reduce the variation of BP value with resting values; individuals were requested to take 10 min rest at sitting position before measuring the BP. Blood pressure was measured by standardized protocols, and hypertension was defined based on the criteria of the Seventh Report of the Joint National Committee on Prevention, Detection, Evaluation, and Treatment of High Blood Pressure. According to this protocol, systolic and/or diastolic blood pressure ≥130/85 mmHg and/or the current use of antihypertensive medication in diabetes diagnosed as hypertension. We used standard electronic B.P. measuring device. Before registering for the study written consent was obtained from each participant in respect to their willingness to take part in the study. Institutional Ethics committee at Jiwaji University Gwalior Madhya Pradesh (India) approved the study protocol. The patients assent was also taken an account before initialization of the study.

Blood sample (3 ml) was collected from each subject. Plasma was separated by centrifuging blood at 8000rpm for 10 min and analyzed for fasting blood glucose. The blood glucose was determined by glucose oxidase-peroxidase method using a kit Monozyme India limited, Ahmadabad (Trinder, 1969). Total cholesterol, Triglyceride and HDL- Cholesterol were estimated by CHOD-PAP (Stockbridge et al., 1989), triglycerides (Fossati & Prencipe, 1982) and HDL- Cholesterol (Lopes-virella et al., 1977) was estimated by spectrophotometric assays employing commercially available kits. LDL and VLDL were calculated from Freidewald’s formula.

### 2.1 Criteria for Metabolic Syndrome (WHO, NCEP-ATPIII and IDF Definition)

The three criteria used for defining MetS are shown henceforth. The WHO definition of 1999 (Alberti et al., 1998) includes impaired glucose tolerance or diabetes and or insulin resistance. The term impaired glucose endurance i.e. impaired fasting blood tolerance; IGT = 7.8—11.0 mmol/l) and or diabetes mellitus; FBG ≥ 6.1 mmol/l), was taken into account as well as two or more of the following components:

- Elevated arterial blood pressure ≥140/90 mmHg.
- Raised plasma triglyceride (≥150 mg/dl).
- Low HDL-cholesterol, (<35 mg/dl for men and <39 mg/dl for women).
- Central obesity (WHR: >0.90 for men and >0.85 for women) and/or BMI (>30 kg/m2).
- Microalbuminurea (urinary albumin excretion rate ≥20 min or albumin: creatinine ratio ≥30 mg/g).

Serum insulin and urine albumin excretion were not measured in our study subjects but the Proteinurea was measured qualitatively by sulphosalisylic acid precipitation test using repeated samples.

### 2.2 NCEP, ATP III Criteria

According to the NCEP, ATP III criteria in 2001 (National Cholesterol Education Program JAMA, 2001) for a person to be defined as having the metabolic syndrome she/he must have combination of any three or more of the following parameters: waist circumference (>102 cm for men and >88 cm for women); plasma triglycerides (≥ 150 mg/dl); HDL cholesterol (< 40mg/dl for men and < 50mg/dl for women); blood pressure (≥ 130/85 mm Hg), and fasting plasma glucose (≥110 mg/dl) were classified MetS group.

### 2.3 New IDF Criteria

According to the IDF definition in 2005 (International Diabetes Federation: The IDF consensus worldwide definition of the metabolic syndrome, 2005) for a person to be defined as having the metabolic syndrome she/he must have central obesity with ethnicity specific values for different groups. We used WC ≥90 cm in men or ≥80 cm in women which is recommended for South Asians (Chobanian et al., 2003) plus any two or more of the following four factors:

- Raised TG levels ≥150 mg/dl (1.7 mmol/l), or specific treatment for this lipid abnormality;
- Reduced HDL-cholesterol <40 mg/dl (1.03 mmol/l) in males and <50 mg/dl (1.29 mmol/l) in females, or specific treatment for this lipid abnormality;
- Raised blood pressure: systolic BP ≥130 or diastolic BP ≥85 mmHg or treatment of previously diagnosed hypertension; and
- Raised fasting blood glucose ≥100 mg/dl (≥5.6 mmol/l) or previously diagnosed diabetes.

The prevalence of MetS using the above criteria was expressed in percentages. Student t test was used for mean of the two groups viz metabolic and non metabolic syndrome group. Kappa (*κ*) statistics was used for finding the agreement between the three definitions. Stepwise binary logistic regression model is used to test the associations between baseline variable and the Metabolic syndrome. For computation of data, software application programs like Microsoft Excel, Sigma Stat, Sigma Direct, and also for logistic regression SPSS trail version were used. The values were tested for significance at P<0.001, P<0.05.

## 3. Results

### 3.1 Characteristics’ of study population

A total of 700 type 2 diabetic participant were recruited, with (n=504) male and (n=196) female. Table 1 is showing the different clinical parameters among male and female group of subject. The mean age of the participant was 54 years and the mean duration of diabetes was 5.6 years with (P≤0.05) significant difference between male and female. When comparison was made between male and female, fasting blood glucose, weight, height, BMI, systolic as well as diastolic blood pressure, pulse, cholesterol were observed to be significant (P<0.001).

**Table 1:** Demographic, anthropometric and biochemical variables of study population

Group	Male (504)	Female (196)	Difference (95% CI)
Age (years)	55±9.5	53±10	2 (.406, 3.5) *
Duration of Disease (years)	6±5	5.2±4	0.8 (.0163, 1.58) *
Fasting (mg/dl)	139.8±57	157.8±65.8	-18 (-27.8,-8.15) **
P.P (mg/dl)	207±83	219±89.3	-12 (-26.01, 2.017)
Waist Cir. (Inches)	38.4±19.4	36.8±21.1	1.6 (-1.687, 4.887)
Weight (kg)	67.6±11.6	61±11.2	6.6 (4.7, 8.4) **
Height (cm)	165.3±8.7	153.6±7.6	11.7 (10.3, 13.09) **
BMI (Kg/m^2^)	24.7±4	25.9±4.5	-1.2 (-1.8, 5.15) **
Systolic pressure (mmHg)	132.7±20	127.2±18.5	5.5 (2.2, 8.7) **
Diastolic pressure (mmHg)	76.7 ±10.5	74.2±10	2.5 (.78, 4.2) *
Pulse rate (/min.)	87.6±11.7	92±12.1	-4.4 (-6.3, 2.4) **
Cholesterol (mg/dl)	155.3 ±50.7	169.3±50.4	-14 (-22.3,-5.6) **
Triglyceride (mg/dl)	134.5±85.3	138.8±61.3	-4.3 (-17.4, 8.8)
HDL-C (mg/dl)	48.7±21.9	49.8±19.2	-1.1 (-4.6, 2.4)
VLDL(mg/dl)	26.4±14.4	27.7±12.2	-1.3 (-3.584, 0.984)
LDL(mg/dl)	90±47.4	98.7±45	-8.7 (-16.4,-0.97) *
T.C/HDL-C (R.R)	3.7±2.05	3.8±1.8	-0.1 (-.428, .228)

Data expressed as Mean ±SD,

* *P*< .05,

** *P*< .001. *Difference is the difference* in the mean or percentage of the variable between males and females

**Figure 1:**
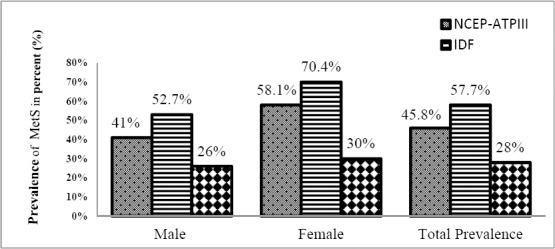
The prevalence of metabolic syndrome among type II diabetes mellitus using NCEP-ATPIII, IDF and WHO criteria

**Figure 2:**
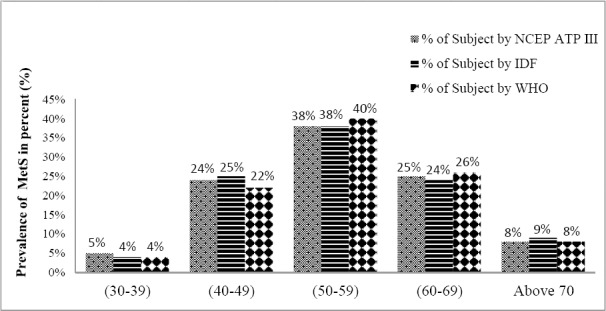
Metabolic syndrome in different age group of subjects

Metabolic syndrome was diagnosed in 321 (45.8%) [95%CI: 42.31-49.69], 404 (57.7%) [95%CI: 54.05-61.37], 196 (28%) [95%CI: 24.67-31.33] participants using the NCEP-ATPIII, IDF and WHO criteria respectively (Figure 1). The prevalence of MetS among male and female were (41% vs 58.1%), (52.7% vs 70.4%), (26% vs 30%) for NCEP, IDF and WHO criteria respectively. Prevalence of metabolic syndrome by different criteria in different Age group of subjects is depicted in Figure 2. The prevalence of metabolic syndrome was found to be highest in age group of 50-59 years with 38%, 38%, 40% based on criteria ATP III, IDF and WHO. It was also observed from figure that (60-69) years and (40-49) years have almost same prevalence of metabolic syndrome. Lowest prevalence was observed in age group of (30-39) years of (5%, 4%, 4%) by employing the respective definitions.

### 3.2 Agreement between the NCEP-ATP III, IDF and WHO Criteria and Venn diagram

The agreement between three definitions of metabolic syndrome was depicted in Table 2. The participants who were classified as having metabolic syndrome by NCEP-ATP III criteria, 95% also met the criteria of IDF. However, 24.5% (99/404) of the subjects identified as positive following IDF classification were not found to have the condition according to NCEP criteria. Good agreement was observed between ATP III and IDF criteria (k = 0.680). Lowest agreement was observed between IDF and WHO criterions for MetS. Of the total participants having metabolic syndrome by IDF criteria, only 43.8% (177 out of 404) met the criteria of WHO.

**Table 2:** Agreement between the NCEP-ATP III, IDF AND WHO Criteria in diagnosing metabolic syndrome

			NCEP ATP III Criteria		Kappa
			Metabolic syndrome		
IDF	Metabolic syndrome	Present	Absent	Total	0.68
	Present	305	99	404	95%CI=(.60-.74)
	Absent	16	280	296	
	Total	321	379	700	

			WHO Criteria		Kappa
			Metabolic syndrome		
IDF	Metabolic syndrome	Present	Absent	Total	0.34
	Present	177	227	404	95%CI=(0.28-0.40)
	Absent	19	277	296	
	Total	196	504	700	

			NCEP ATP III Criteria		Kappa
			Metabolic syndrome		
WHO	Metabolic syndrome	Present	Absent	Total	0.54
	Present	182	14	196	95%CI=(.47-.61)
	Absent	139	365	504	
	Total	321	379	700	

In contrast, the subjects without the metabolic syndrome according to the IDF criteria, 9.6% were positive following the WHO criteria (k = 0.34). This may have been due to the WHO definition of MetS which do not include any subjects without impaired glucose regulation. In those subjects who were classified as having the metabolic syndrome according to WHO criteria, 92.8% satisfied the ATP III criteria. Conversely the participants without the syndrome according to the WHO criteria 27.5% met the criteria of ATP III. The agreement between WHO and ATP III is moderate (k = 0.54) but higher than the observed agreement between WHO and IDF criteria.

**Figure 3:**
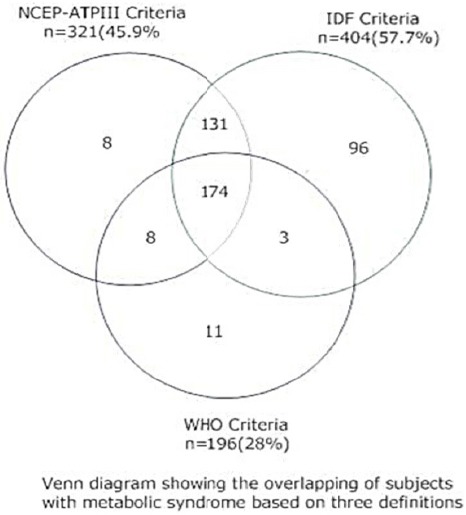
Venn diagram showing the overlapping of subjects with metabolic syndrome based on three definitions

Venn diagram has been shown in Figure 3 to verify the agreement result. Here also the number of subjects with MetS in NCEP-ATPIII & IDF was 305 whereas by NCEP-ATPIII and WHO criteria the number with MetS is 182. The number of subjects overlapped between IDF and WHO criteria was 177. The minimum number of overlapped metabolic syndrome subject was found in IDF and WHO criterion.

### 3.3 Distribution of Clinical Data among Metabolic and Non Metabolic Groups of Type 2 Diabetes Classified according to NCEP ATP III, IDF & WHO Criteria

According to NCEP criteria the clinical data among two groups MetS and Non MetS of type 2 diabetes is given in Table 3. Taking together male and female, waist circumference, blood pressure (systolic as well as diastolic pressure), triglyceride & HDL-C were found to be Significant (P≤0.001) in metabolic syndrome group compared with non metabolic syndrome. The age group in male as well as in female was not found to be significant when classified with NCEP definition. The table could also observe an association of MetS with type 2 diabetes characterized by significant elevated level of triglyceride, central obesity, fasting blood glucose etc.

**Table 3:** Comparison of clinical data among two groups (Metabolic and Non Metabolic) of type 2 diabetic subjects according to NCEP Criteria

		Subjects with Metabolic Syndrome	Subjects without Metabolic Syndrome	Significance
**Male**	N	207	297	
	Age (year)	55.7±8.8	54.2±9.9	P=.098
	Waist Circumference (Inches)	37.8±3.2	35.7±3.4	P=<0.001* *
	SBP (mmHg)	142.6±14.7	126.5±20.9	P=<0.001* *
	DBP (mmHg)	82.2±10.4	73.2±9.7	P=<0.001* *
	Duration of disease (year)	6.1±5	5.7±5.4	P=.841
	Triglyceride (mg/dl)	159.6±77.6	114.9±56	P=<0.001* *
	HDL-C (mg/dl)	42.6±19.6	52.8±21.7	P=<0.001* *
	Fasting (mg/dl)	151.3±48.6	134±60.8	P=<0.001* *

**Female**	N	114	82	
	Age (year)	52.8±8.9	53.4±10.3	P=.664
	Waist Circumference (Inches)	39.5±4.44	33.1±3.3	P=<0.001* *
	SBP (mmHg)	134.2±14	118.5±15.4	P=<0.001* *
	DBP (mmHg)	77.3±10	70.3±7.3	P=<0.001* *
	Duration of disease (year)	6±5.6	4.6±3.6	P=.009*
	Triglyceride (mg/dl)	158±60.6	119.8±54.1	P=<0.001 * *
	HDL-C (mg/dl)	45.3±17.1	56.4±20	P=<0.001* *
	Fasting (mg/dl)	163.9±58.9	149.8±74.3	P=.045 *

BMI: Body mass index (kg/m^2^) SBP: Systolic Blood Pressure (mmHg) DBP: Diastolic Blood Pressure (mmHg) HDL-C:High density Lipoprotein cholesterol

Data were expressed as mean ±standard deviation. Student t test was used for mean of the two groups. Levels of significance were represented in the form of

(**) P<0.001,

(*) P<0.05 when compared with non metabolic syndrome subjects.

**Figure 4:**
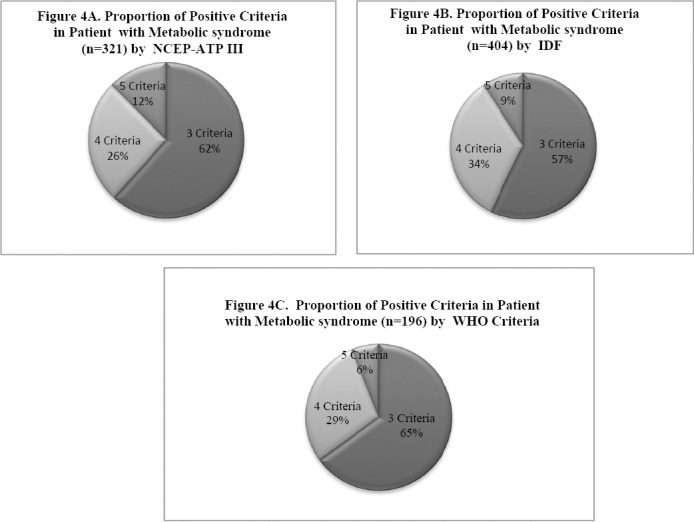
Different proportions of positive criteria in MetS subjects using NCEP-ATPIII, IDF & WHO criteria

Differences in proportion of positive criteria in MetS with type 2 diabetes diagnosed by NCEP, IDF & WHO criteria are shown in Figure 4 (4A showing NCEP definition, 4B showing IDF & 4C showing WHO criteria). The pie chart is showing differences in the proportion of positive criteria in all three respective definitions. Interestingly, we showed highest percentage of proportion in 3 criteria group was in WHO, leads to the lower combination of glucose parameter which is the mandatory variable in this definition. The three criteria group is showing more prevalent than combination of 4 or 5 criteria. Almost half of the percent of the 3 criteria recorded in combination of 4 criteria group. As the metabolic syndrome parameters are more closely interacted with each other. Blood pressure, triglyceride and HDL-C were commonly viewed in this combination (data not shown)

The comparison of anthropometric characteristics and metabolic variables of groups for MetS according to IDF and WHO criteria are shown in Table 4 & 5. The table was grouped as above mentioned, the IDF criteria showed 404 participants were having MetS while 196 subjects were positive by WHO criteria. In males, the waist circumference, blood pressure, triglyceride, HDL-C level were found to be more significant (P≤0.001) in both diagnostic criterion except the age and fasting glucose showed a non significant statistical analysis while applying to the WHO criteria. The WHO criteria is more strict on the glucose level (mandatory) in individuals before adding to another variable in the definition. In spite of that the study did not show a significant value while comparing with definition of IDF, waist circumference is the obligatory variable in diagnosing MetS. The study observed more influence of waist circumference in identifying elevated level MetS by this criterion regardless to the fasting blood glucose level in WHO criteria. In females, metabolic syndrome classified by IDF and WHO criteria establish as similar appearance or showing alike distribution of variable between the two groups except age and fasting glucose level but had showed glucose to be significant (P≤0.05) identified in IDF criteria.

**Table 4:** Comparison of clinical data among two groups (Metabolic and Non Metabolic) of type 2 diabetic subjects according to IDF Criteria

		Subjects with Metabolic Syndrome	Subjects without Metabolic Syndrome	Significance
**Male**	N	266	238	
	Age (year)	55.8±8.9	53.9±10	P=.024*
	Waist Circumference (Inches)	38±3.1	34.7±3.6	P=<0.001 * *
	SBP (mmHg)	139±19	125.5±18.6	P=<0.001 * *
	DBP (mmHg)	79.5±9.7	73.5±10.5	P=<0.001 * *
	Triglyceride (mg/dl)	150±77	112±56	P=<0.001 * *
	HDL-C (mg/dl)	44±20	54±21	P=<0.001 * *
	Fasting (mg/dl)	150±51	130±62	P=<0.001 * *

**Female**	N	138	58	
	Age (year)	52.5±9.6	54.4±10.9	P=.226
	Waist Circumference (Inches)	36.3±3.8	33±4	P=<0.001 * *
	SBP (mmHg)	132±17.7	116.4±15.9	P=<0.001 * *
	DBP (mmHg)	75.9±10.3	69.9±8.02	P=<0.001 * *
	Triglyceride (mg/dl)	149±62	115±53	P=<0.001 * *
	HDL-C (mg/dl)	46±17	59±21	P=<0.001 * *
	Fasting (mg/dl)	166±59	139±77	P=.008 *

Data were expressed as mean ±standard deviation. Student t test was used for mean of the two groups. Levels of significance were represented in the form of

(**) P<0.001,

(*) P<0.05 when compared with non metabolic syndrome subjects.

**Table 5:** Comparison of clinical data among two groups (Metabolic and Non Metabolic) of type 2 diabetic subjects according to WHO Criteria

		Subjects with Metabolic Syndrome	Subjects without Metabolic Syndrome	Significance
**Male**	N	135	369	
	Age (year)	54.8±9.3	54.3±9.2	P=.590
	BMI (kg/m2)	26.6±3.6	24.1±3.5	P=<0.001* *
	SBP (mmHg)	146.7±19.1	127.5±19.3	P=<0.001* *
	DBP (mmHg)	83.9±10	74±10.1	P=<0.001* *
	Triglyceride (mg/dl)	168.5±11.8	123±54.8	P=<0.001* *
	HDL-C (mg/dl)	41.4±23	52.1±25.7	P=<0.001* *
	Fasting (mg/dl)	147.4±52	139±52	P=0.109

**Female**	N	61	135	
	Age (year)	53.8±8.2	53.2±9.6	P=.673
	BMI (kg/m2)	27.1±4.9	25±4.3	P=0.003*
	SBP (mmHg)	138±20.1	127.3±16	P=<0.001 * *
	DBP (mmHg)	79±10.8	74±9.6	P=<0.001* *
	Triglyceride (mg/dl)	174.2±66	137.2±64.9	P=<0.001* *
	HDL-C (mg/dl)	39.5±15.7	50±22.5	P=0.001 * *
	Fasting (mg/dl)	166.9±61	157.2±70	P=0.352

Data were expressed as mean ±standard deviation. Student t test was used for mean of the two groups. Levels of significance were represented in the form of

(**) P<0.001,

(*) P<0.05 when compared with non metabolic syndrome subjects.

### 3.4 Prevalence of Metabolic Syndrome for Patients with T2DM (Stepwise Binary Logistic Regression Model)

To determine the independent predictors of the prevalence of MetS a forward stepwise binary logistic regression was performed and shown in Table 6 to test 6 predictor variables: fasting blood glucose, systolic and diastolic blood pressure, waist circumference, triglyceride, HDL-cholesterol. We used NCEP-ATPIII criteria for the diagnosis of MetS. This is the only criterion definition that reflects equal importance to the every variable and showed a good agreement between the different criteria used. The dependent variable was the MetS (present, absent). A total of 700 type 2 diabetic subjects were recruited with 321 MetS and 379 were non MetS for this analysis. Higher fasting blood glucose (RP 1.008 (95% CI: 1.005-1.011), systolic blood pressure (RP 1.047 (95% CI: 1.034-1.060), diastolic (RP 1.059 (95% CI: 1.035-1.083), Triglyceride (RP 1.014 (95% CI: 1.011-1.018) low HDL-C (RP 0.969 (95% CI: 0.960-0.979).waist circumference (RP 1.232 (95% CI: 1.162-1.307) significantly increased the prevalence of metabolic syndrome.

**Table 6:** Risk factors related to prevalence of metabolic syndrome in patients with T2DM (stepwise binary logistic regression

Variables	Odds ratio (95% CI)
Fasting blood glucose (mg/dL)	1.008(95% CI: 1.005-1.011) **
Systolic BP (mmHg)	1.047(95% CI: 1.034-1.060) **
Diastolic BP (mmHg)	1.059(95% CI: 1.035-1.083) **
Triglyceride (mg/dL)	1.014(95% CI: 1.011-1.018) **
HDL-C (mg/dL)	0.969(95% CI: 0.960-0.979) **
Waist Circumference (Inches)	1.232(95% CI: 1.162-1.307) **

*P < .05,

** *P* < .001.

Our study assessed that the prevalence of MetS was highest by using IDF definition. Taking this result into account we tabulated the prevalence of individual component of MetS that could elucidate the higher influence on Indian population which may be targeted for reducing the prevalence rate. Table 7 showed the prevalence of metabolic syndrome by IDF definition (n=404) in which 266 males and 138 females. 83% of males and 94% of the females’ subjects had high waist circumference. The prevalence of blood pressure in metabolic syndrome subjects is 69% in which 75.5% of males and 57% of female’s subjects had high blood pressure as defined by IDF. The overall percentage of high triglyceride and low high density lipoprotein in metabolic syndrome subjects was 44% and 59% respectively. In male it was 44% and 56% but in female it was 45% and 66% respectively.

**Table 7:** Frequency of waist circumference, high blood pressure, elevated triglyceride, low HDL cholesterol in metabolic syndrome subjects diagnosed by IDF Criteria (n=404) out of 700 type 2 diabetic subject.

Characteristics		Male	Female
Metabolic syndrome	404	266	138
Waist circumference(Inches)	351(87%)	221(83%)	130(94%)
Blood Pressure(mmHg)	280(69%)	201(75.5%)	79(57%)
High Triglyceride(mg/dl)	179(44%)	117(44%)	62(45%)
Low HDL-C(mg/dl)	239(59%)	148(56%)	91(66%)

## 4. Discussion

In this study we assessed the prevalence of MetS in an urban central Indian diabetic population using the World Health Organization (WHO), National Cholesterol Education Program-Adult Treatment Panel-III (NCEP- ATPIII) and International Diabetes Federation (IDF) criteria simultaneously and in order to assess the true defining MetS criteria by using the Agreement analysis in central Indian type 2 diabetic populations. The study also confers the ability to identify cardiovascular risk factors in terms of Metabolic and non metabolic syndrome group of subject in males and females. These three definitions consist of essential components like glucose intolerance, Hypertension, obesity, and dyslipidemia however they are having different cut-off for each parameter and also exhibited different combinations in variable to diagnosing MetS. The International Diabetes Federation has recently provided a definition more applicable in view of different ethnic populations by providing a range for increased waist circumference, which was lower for certain racial groups which is the most important one for Indian populations because we have different ethnic population from other Asian country (WHO Western Pacific Regional Office, 2000).

MetS appears to be quite common in urban Indian T2DM with an estimated prevalence of 57.7%, 45.9%, 28% by IDF, NCEP-ATP III and WHO criteria (Figure 1). Since the higher and lower prevalence may be due to different cutoffs of markers of abdominal obesity and also some other variables. Kengne et al., in 2012 reported similar observation of prevalence study on MetS. This study showed higher prevalence of MetS by using IDF criteria in comparision with NCEP-ATP III criteria among type 2 diabetic sub-Saharan Africans. More than 10% of differences have been seen in this study.

We also find the high degree of concordance between NCEP-ATPIII & IDF criteria which could be explained by the fact that the two definitions use the same five diagnostic components and apart from WC (essential component in IDF), the remaining criteria are nearly identically defined. The prevalence of metabolic syndrome was found to be highest in age group of 50-59 years with 38%, 38%, 40% based on criteria of ATP III, IDF and WHO as shown in Figure 2. We observed a good agreement of Kappa value between ATP III and IDF criteria (k = 0.680). Ahmed at al. (2012) reported a similar trend of agreement as observed in our study. The study concluded highest Kappa value agreement between the IDF & NCEP-ATPIII definition (k=0.728)

Least agreement was seen while comparing between IDF and WHO definitions (k = 0.34). Table 3, 4 & 5 symbolized the comparison of clinical data among two groups (Metabolic and Non Metabolic) of type 2 diabetic subjects according to different criteria used for the study.. It was quite interesting to see the significances between the two groups respective of different variable. The metabolic syndrome with type 2 diabetic subjects diagnosed by the three definitions showed a well significance except for age and glucose level in females when compared with the non metabolic ones.

The affect of independent predictors’ of the prevalence of MetS a forward stepwise binary logistic regression was performed shown in the Table 6. Accurate information regarding the prevalence of MetS and associated risk factors in people with diabetes is important for the prevention or delaying of complications including macro and microvascular disease. This study elucidates clustering of cardiovascular risk factors, and their interaction between the variables in metabolic syndrome of type-2 diabetic patients in Gwalior Chambal region of Madhya Pradesh (India). In a developing country like India, increasing urbanization and lifestyle changes have led to an increased incidence of diabetes (Ramachandran et al., 2008). Though Limited information is available about the prevalence of the metabolic syndrome in type 2 diabetic patients in developing nations including India. Knowledge of the variables influencing the development of the syndrome may be utilized in interventions that could favorably alter its prevalence.

Consistent with prior studies in diabetic and non-diabetic populations (Ford et al., 2002; Azizi et al., 2003; Ford & Giles, 2003) the present study found similarly increasing prevalence of MetS with increasing age in a diabetic population (Data unpublished). There are several studies reporting prevalence of the MetS in this T2DM population more than double the prevalence in the general populations (Ford, 2002; Azizi et al., 2003; Bonora et al., 1998; Rantala et al., 1999; Lee et al., 2004; Jaber et al., 2004; Meigs et al., 1997; Park et al., 2003; Florez et al., 2005). It is estimated that a large majority of patients with type 2 DM or impaired glucose tolerance have the metabolic syndrome (Isezuo et al., 2005).

In a study of heart disease among the NHANES III participants (Alexander et al., 2003) the excess prevalence of coronary heart disease attributable to MetS and/or diabetes was found to be 37.4% in the group with metabolic syndrome without diabetes and 50.3% in the group with both metabolic syndrome and diabetes. In this regard it is of particular concern that two-thirds of patients with T2DM actually met the criteria of the MetS despite being treated for diabetes, hypertension and dyslipidaemia, with most subjects satisfying the waist circumference, blood pressure and glycemic criteria. Hence it implies the importance of prevalence study in type 2 diabetic subjects.

The present study corroborates with the observations of other studies (Ashraf et al., 2006; AlSaraj et al., 2009; Titty et al., 2008; Isezuo et al., 2005). Our study based figure is lower than the values reported by the Brazilian, Chinese, Italian, British and Scandinavian studies (Bonora et al., 1998; Costa et al., 2004; Lee & Tsai, 2002; Ilanne-Parikka et al., 2004). Surana et al., in 2008 observed the Prevalence in type 2 diabetes in Mumbai as 77.2%. When compared with other studies our study showed less prevalence may be due to the unequal distribution of selected subjects and also we recruited those subjects who were in service among males and most of the females were house wives. We reported that 64.0% of the study patients had dyslipidemia by NCEP criteria (data not shown). Obesity, hypertension, and dyslipidemia were all significantly more common among women in our study (Table 7). Prevalence rates in various studies from around the world show considerable variation. The differences in diagnostic criteria for this syndrome are partially responsible for variations in the reported prevalence among different studies (Bonora et al., 1998; Rantala et al., 1999).

Our study also provides the first estimates of the prevalence of metabolic syndrome in an urban central Indian diabetic population. Our study demonstrated prevalence of metabolic syndrome was higher among women as compared to men. Osuji et al. (2012) in his study reported prevalence of MetS dominanted in female subjects as compared to male among type 2 diabetic Nigerian. Women had a higher prevalence of low HDL and central obesity. This could partially be attributed to the lower cut-off for waist circumference and higher cut-off for HDL in women as compared to men. Therefore, probably more women were classified as having central obesity or low HDL. Men were more likely to have hypertension and hypertriglyceridemia. The prevalence of waist circumference, blood pressure and low HDL-C accordingly to IDF definition for metabolic syndrome group with diabetic subjects was 87%, 69% and 59%, (Table 7). Adults who have type 2 diabetes, the presence of metabolic syndrome is associated with a fivefold increase in CV risk independent of age, sex, smoking status, and glycated hemoglobin (HbA1c) (Bonora et al., 2004). Therefore, it is imperative that aggressive therapy be aimed at controlling hyperglycemia, dyslipidemia and hypertension. According to IDF definition hypertension was quietly higher in males as compared with female while the waist circumferences were dominated in female group. The features of MetS continue to be present in many subjects with diabetes despite treatment for elevated glucose levels and other cardiovascular risk factors. This emphasizes the need for an aggressive multifactorial intervention in the management of cardiovascular risk in subjects with T2DM (Gaede et al., 2003).

Several limitations of our study should be recognized. First, our experimental design was cross-sectional; and hence we could not predict the complications but only suggest that the best criteria suitable for the diagnosis of MetS and could be an indicator of cardiovascular risk. We have not included complete history of medicines and lifestyle in this study that could be one of the limitations in evaluating our result and its prevalence. Our study excluded serum insulin resistance and urinary albumin excretion in the respective definitions. The present analysis is limited in its ability to elucidate causal relationships between risk factors and MetS. But the newness in our study to define the MetS criteria for the type 2 diabetic belonging to the central region of India. The study clearly shows NCEP-ATPIII and IDF definition may be suitable for identifying the high risk population in diabetic subjects. This is the first study showing interaction between these definitions among type 2 diabetic subjects. We also recognize the WHO definition as lower and may be could not reach the appropriate level of identification. Therefore, the study recommend the IDF and NCEP-ATPIII as a best choice and also supported by previous studies. These subjects should be paid attention in term of hypertension, obesity and dyslipidemia for the risk factor for the development of CVD The annual assessment of patients with diabetes with an appropriate definition should be included in calculation of MetS score so that those who have particularly high cardiovascular risk can be targeted for especially aggressive risk factor management.

## 5. Conclusion

In conclusion, our study demonstrates that metabolic syndrome is extremely common among diabetic patients, especially by IDF criteria and the individual factors which may responsible for the development of cardiovascular complications. Therefore it is important for those caring for people with diabetes to be aware of whether their patients also meet the criteria for MetS. Overall, metabolic syndrome can serve as a simple clinical approach to identify persons for intervention to reduce both CVD and type 2 DM.
